# Intra-Species Bacterial Quorum Sensing Studied at Single Cell Level in a Double Droplet Trapping System

**DOI:** 10.3390/ijms140510570

**Published:** 2013-05-21

**Authors:** Yunpeng Bai, Santoshkumar N. Patil, Steven D. Bowden, Simon Poulter, Jie Pan, George P. C. Salmond, Martin Welch, Wilhelm T. S. Huck, Chris Abell

**Affiliations:** 1 Department of Chemistry, University of Cambridge, Lensfield Road, Cambridge CB2 1EW, UK; E-Mails: ybai@kth.se (Y.B.); patil.santosh.n@gmail.com (S.N.P.); jp500@cam.ac.uk (J.P.); wtsh2@cam.ac.uk (W.T.S.H.); 2 Department of Biochemistry, University of Cambridge, Tennis Court Road, Cambridge CB2 1QW, UK; E-Mails: bowdensd@gmail.com (S.D.B.); sp462@cam.ac.uk (S.P.); gpcs2@cam.ac.uk (G.P.C.S.); mw240@cam.ac.uk (M.W.); 3 Radboud University Nijmegen, Institute for Molecules and Materials, Heyendaalseweg 135, Nijmegen 6525 AJ, The Netherlands

**Keywords:** droplet-based microfluidics, bacterial quorum sensing, single cell

## Abstract

In this paper, we investigated the intra-species bacterial quorum sensing at the single cell level using a double droplet trapping system. *Escherichia coli* transformed to express the quorum sensing receptor protein, LasR, were encapsulated in microdroplets that were positioned adjacent to microdroplets containing the autoinducer, *N*-(3-oxododecanoyl)- l-homoserine lactone (OdDHL). Functional activation of the LasR protein by diffusion of the OdDHL across the droplet interface was measured by monitoring the expression of green fluorescent protein (GFP) from a LasR-dependent promoter. A threshold concentration of OdDHL was found to induce production of quorum-sensing associated GFP by *E. coli*. Additionally, we demonstrated that LasR-dependent activation of GFP expression was also initiated when the adjacent droplets contained single *E. coli* transformed with the OdDHL synthase gene, LasI, representing a simple quorum sensing circuit between two droplets.

## 1. Introduction

Quorum sensing is a form of cell-to-cell chemical communication in which bacteria produce, release, and respond to extracellular signaling molecules [1]. When the extracellular concentration of a signaling molecule reaches to a threshold, the response of a cell leads to changes in gene expression. Quorum sensing is regarded as a cell density dependent phenomenon where a group of bacteria can detect cell population and coordinate collective behavior such as virulence [2] and biofilm formation [3]. Quorum sensing was initially studied on clonal populations under laboratory conditions; however, the natural environments where bacterial populations live are complicated and bio-diverse [4]. Bacterial quorum sensing is influenced by other factors such as mass transport, confinement and degradation [5,6]. Furthermore, quorum sensing occurs even between small numbers of cells, which is important for survival and growth of cells in early biofilm formation, or early infection [7].

Advances in experimental methods have facilitated the study of individual parameters in regulating bacterial quorum sensing at single cell level, revealing important factors that may be masked in large populations. Carnes *et al*. and Boedicker *et al*. respectively showed that quorum sensing initiation by single *Staphylococcus aureus* or *Pseudomonas aeruginosa* bacteria is possible if they are confined in a tiny space [7,8]. The constructed microstructure provides a confinement that restricts the diffusion of signaling molecules and results in bacteria responding as if present at a high cell-density. These experiments employed quorum sensing bacteria to set up a closed self-inductive quorum sensing circuit. However, to our best knowledge the communication between individual confined bacteria has not been reported.

In this paper, a droplet-based microfluidic system is used to confine single bacterium for studying the influence of diffusion and confinement on quorum sensing. Microdroplets separated by inert oil and surfactant monolayers can be used as micro-reactors or micro-wells for the separation and encapsulation of single cells [9–11], enabling continuous monitoring of biological events at the single cell level [12–14]. It provides a high-throughput method to study cells quantitatively. Recently, we developed a microfluidic method for efficiently trapping two different droplets together and studied the diffusion of hydrogen peroxide across the droplet interface [15]. Here we develop this technique such that two cells encapsulated separately in two adjacent droplets can “talk” to each other by means of diffusion of signaling molecules across droplets. Initially one droplet contained the signaling molecule *N*-(3-oxododecanoyl)- l-homoserine lactone (OdDHL), and the other an OdDHL sensing *E. coli*. Subsequently a droplet containing OdDHL secreting *E. coli* was trapped adjacent to one containing OdDHL sensing *E. coli*, and the communication between two bacteria observed by monitoring the production of GFP in *E. coli*.

## 2. Results and Discussion

One of the technical challenges for cell-cell communication studies using encapsulated droplets at the single cell level is how to efficiently trap double droplets in microfluidics. Previously, we reported an approach to pair two different droplets with 70% efficiency [15]. The droplets were trapped in an array, where each droplet pair could be imaged using fluorescence microscopy. For the present study, two types of droplets were generated: one contained the signaling molecule (OdDHL) or *E. coli* expressing LasI, which was used as the source of the chemical signal to influence the behavior of LasR-expressing *E. coli* encapsulated in the other droplet. The *E. coli* reporter assay system is based on the natural quorum sensing system of *Pseudomonas aeruginosa*.

**Figure 1:**
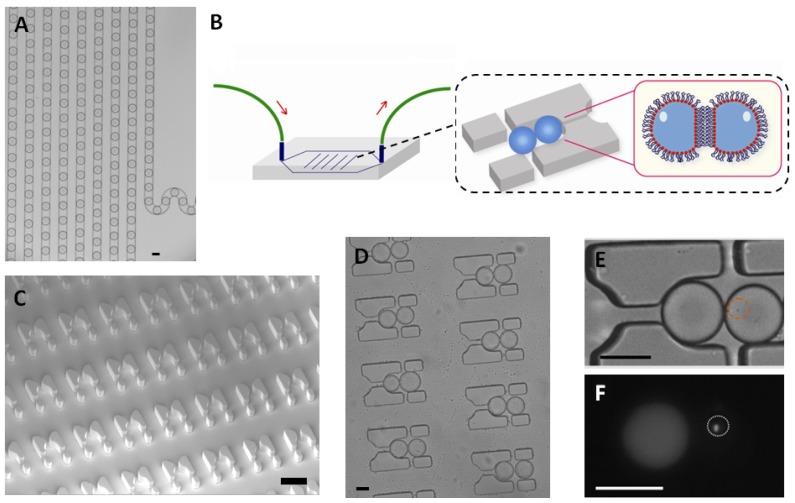
Experimental setup. (**A**) Generation of a monodisperse emulsion with a microfluidic droplet production device. Aqueous droplets (65 picoliter) containing a single bacterium or signaling molecules were generated in fluorinated oil using a flow-focusing configuration; (**B**) Illustration of the injection of a prepared emulsion into the trapping device. Double droplet pairs were confined in traps where a droplet interface bilayer formed between droplets; (**C**) The scanning electronic microscopy (SEM) image of arrayed traps in the trapping device, which was fabricated using the standard soft lithography [16]; (**D**) Double droplets were trapped in the microfluidic device; (**E**) The bright field image of the two trapped droplets. The left droplet contained rhodamine B isothiocyanate-dextran labeled microbeads (500 nM) with 10 μM OdDHL, and the right droplet encapsulated LasR-expressing *E. coli* (indicated by the orange circle) which contained a plasmid (pSB1014) that caused constitutive expression of red fluorescent protein (RFP) in the presence of arabinose; (**F**) The red fluorescence image showed the LasR-expressing *E. coli* (indicated by the white circle) due to the expressed RFP. The construction of plasmid followed the previous work [17,18]. Bar scales: (**C**) 60 μm, (**D**) 40 μm, (**E**,**F**) 50 μm.

First, single LasR *E. coli* were trapped in a 65 picoliter droplet (Figure 1A). The production of droplets was performed in a flow-focusing microfluidic chip, and the size of droplets was tuned to around 50 μm under conditions where the encapsulation of a single bacterium follows a Poisson distribution (approximately 72% of droplets were empty, 18% of droplets contained a single bacterium and the remaining 10% contain two or more cells, calculated from droplets, data not shown). Droplets were transported into the trapping device through polyethylene tubing, wherein two kinds of droplets were trapped together (Figure 1C–E). The time of the formation of the double droplet pairs was taken as the initial time (*t* = 0) for droplet diffusion experiments. To distinguish between the two droplets, rhodamine B isothiocyanate-dextran labeled microbeads were added into the droplet containing the signaling molecule and its paired droplet contained *E. coli* bearing the plasmid (pSB1014) that caused constitutive expression of RFP (Figure 1F). Once two droplets touched each other, a droplet interface was established. Small molecules diffuse across the interface, driven by a concentration gradient. This concentration gradient was established by either compartmentalizing signaling molecules or by entrapping a single bacterium that secrete signaling molecules into the droplet adjacent to the droplet containing the quorum sensing bacteria. In this experiment, OdDHL was used as the signaling molecule, as it is also produced by the LasI-expressing *E. coli*. It is known that droplets trapped in the PDMS device will shrink due to the evaporation of water [12]. To avoid this, the arrayed droplets were placed in a water-saturated environment, which made it possible to quantify bacterial signaling over a longer duration.

**Figure 2:**
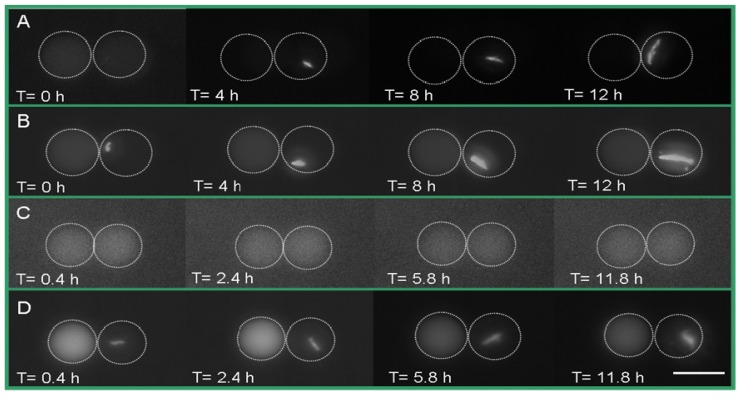
LasR-expressing *E. coli* responding to the signaling molecule, OdDHL and the negative control experiment. (**A**) The left droplet contains rhodamine B isothiocyanate-dextran (500 nM) and OdDHL (1 μM). The right droplet contains LasR-expressing *E. coli*. Fluorescent images show an increase in green fluorescence protein (GFP) fluorescence in bacteria as a function of time; (**B**) RFP production in the same compartmentalized single bacterium shown in A; (**C**) The left droplet contained rhodamine B isothiocyanate-dextran (500 nM) without OdDHL and the right droplet contained LasR-expressing *E. coli*. No production of GFP was observed in the green fluorescent images; (**D**) RFP production in the same single bacterium shown in C. The white circles indicate the boundary of the droplets. Scale bar: 50 μm.

The GFP-based *N*-acyl homoserine-lactone (AHL) sensor system has been used to detect bacterial communication [19]. Here, *E. coli* transformed with a gene for GFP under the control of LasR and a second plasmid expressing the red fluorescent protein, mCherry, were detected to study the response of bacteria to signaling molecules using two filters simultaneously. Green fluorescence protein (GFP) will be expressed if LasR *E. coli* senses the presence of OdDHL. As shown in Figure 2A, droplets containing 1 μM of OdDHL and 500 nM of rhodamine B isothiocyanate-dextran were trapped adjacent to droplets containing *E. coli* expressing the LasR receptor. The simultaneous detection of red and green fluorescence was carried out over 12 h. The production of GFP by LasR-expressing *E. coli* started at around 2.4 h, and continued until 12 h (Figure 2A). This indicated that OdDHL had diffused from adjacent droplets into the droplets containing bacteria and triggered LasR-dependent GFP production. This observation was further confirmed by a negative control experiment, where LasR *E. coli* did not produce GFP in the absence of OdDHL in the adjacent droplet (Figure 2C). The same bacteria (Figure 2B,D) expressed mCherry, showing they were viable throughout the experiment. This result demonstrated that LasR *E. coli* detected the OdDHL diffusing from the other trapped droplet and responded to it by expressing GFP. It should be noted that the single *E. coli* divided in the droplet indicating that droplets had provided a good environment for cell growth.

**Figure 3:**
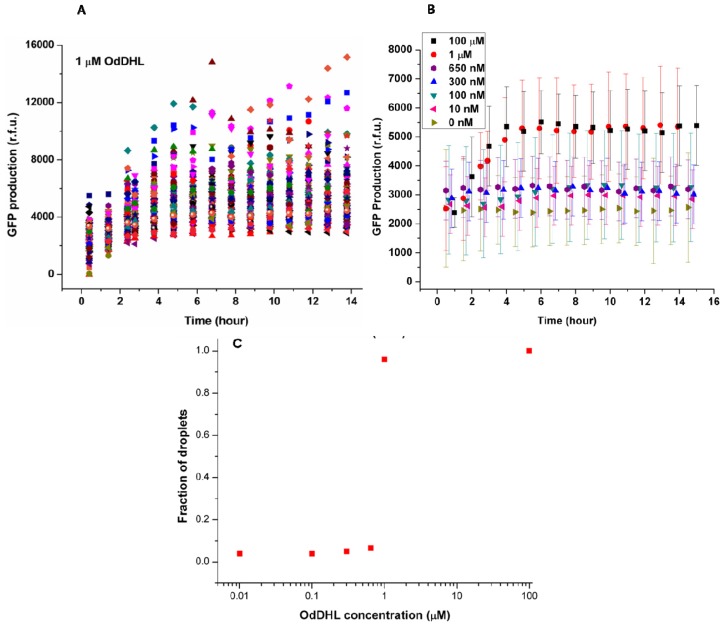
(**A**) Kinetics of GFP production by LasR-expressing *E. coli* cells encapsulated in microdroplets trapped adjacent to droplets containing 1 μM OdDHL. The graph shows the change of fluorescence intensity from bacteria, which were measured at hourly intervals, up to 14 h. In this experiment, 160 droplets containing bacteria (out of a total of 1050 droplet pairs) were analyzed; (**B**) The time course of GFP production by LasR-expressing *E. coli* per droplet at different concentrations of OdDHL in the neighbouring droplet. The data of each concentration was averaged from hundreds of single cells; (**C**) Fraction of droplets with *E. coli* induced by different concentrations of OdDHL.

The concentrations of OdDHL were varied from 10 nM to 100 μM to find the threshold for quorum sensing of LasR *E. coli* in this system. In each experiment, hundreds of individual bacteria were analyzed by taking pictures of droplets located in different areas of the trapping device and measuring the fluorescence intensity from bacteria. Figure 3A shows the change of green fluorescence intensity in *E. coli* with 1 μM OdDHL in the neighboring droplet. Each dot represents GFP production measured in the single droplet. It was clear that individual bacteria expressed GFP to different extents. The significant differences in GFP production may be attributed to different plasmid copy numbers in *E. coli*, different cell division cycle stages of trapped cells, and different initial occupancies of bacteria due to the Poisson distribution. The onset of GFP production was around 1.5 h after droplet entrapment. This delay includes the time required for diffusion of the signaling molecule across the droplet bilayer, and the synthesis and maturation of the protein fluorophore.

The quorum sensing response in *E. coli* depends on the diffusion of the signaling molecule, which depends upon its concentration in the adjacent droplet. This dependency is demonstrated by the averaged response data as a function of varying concentrations of OdDHL (from 10 nM to 100 μM, Figure 3B). Above 1 μM, the production of GFP quickly started at around 1.5 h, and reached a maximum after 4 h. Both 100 μM and 1 μM OdDHL experiments showed a similar trend. In comparison, the fluorescence intensity was almost constant and low during the experiment for OdDHL concentrations from 10 to 650 nM, and no green fluorescence was observed, which suggested insufficient OdDHL to initiate the LasR-expressing *E. coli* to trigger a response. The absence of GFP production in the absence of OdDHL suggests that the plasmid-based reporter system was under sufficiently tight control. To further understand the influence of OdDHL concentration on bacterial signaling in the engineered *E. coli*, we calculated the fraction of droplets (*F* = *N*_QS_/*N_E. coli_*) showing quorum sensing. *N*_QS_ is the number of droplets containing *E. coli* that responded to OdDHL, as measured using green fluorescence images. N*_E. coli_* is the total number of droplets containing *E. coli*, that were counted in the red fluorescence images. The results obtained after 14 h indicate that there was a well-defined concentration threshold above which the sensor strain was able to detect and respond to the signaling molecule (Figure 3C). When 1 μM or more OdDHL was contained in droplets, 95% of compartmentalized bacteria in droplets were found to be producing GFP. However, if the concentration of OdDHL was lower than 1 μM, the fraction dropped to nearly 5%. This result indicated that even separated apart in different parts of the trapping device, most bacteria can carry on quorum sensing in a similar and collective manner.

**Figure 4:**
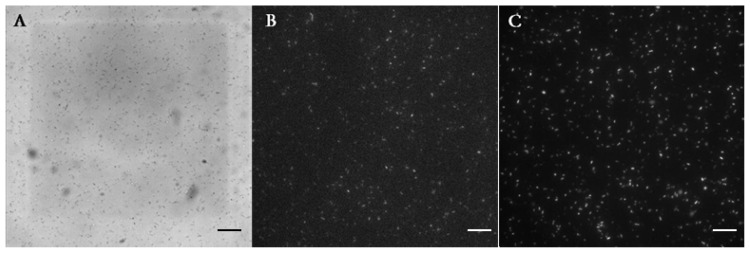
Quorum sensing of LasR *E. coli* in bulk aqueous phase. (**A**) The bright field image of LasR *E. coli* mixed with 1 nM OdDHL in bulk; (**B**) The green fluorescent image shows the expression of GFP in bacteria; (**C**) The red fluorescent image shows the same bacteria that expressed RFP.

We tested the threshold of same bacteria in bulk aqueous phase where the suspension of LasR *E. coli* was directly mixed with the solution of OdDHL, and found that 1 nM OdDHL caused quorum sensing of LasR *E. coli* (Figure 4). The thousand fold difference between the thresholds observed in homogenous solution and using the separated droplets presumably reflects hindered diffusion of OdDHL by the heterogeneous phases and the layers of surfactants. The threshold found in this paper (1 μM) was lower than reported previously (10 μM) [20] using a system where autoinducer was delivered through a PDMS membrane.

**Figure 5:**
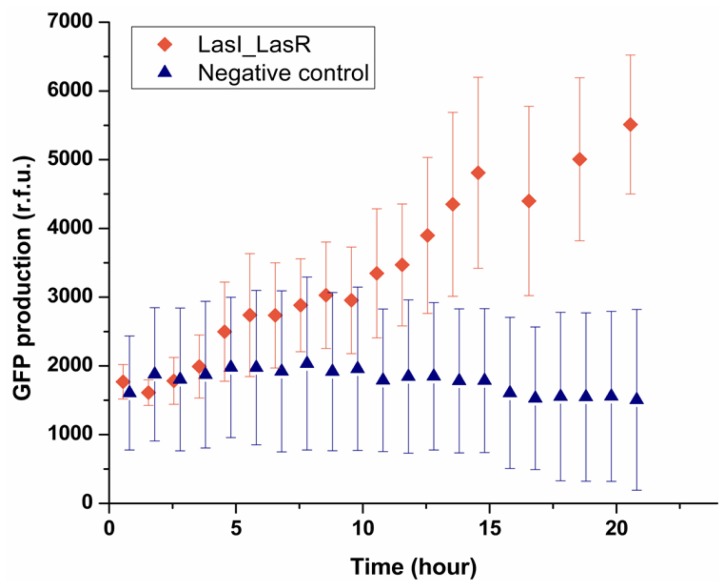
LasR-dependent GFP production triggered by diffusion of OdDHL secreted by LasI-expressing *E. coli* encapsulated in the droplet contacting the droplet containing LasR-expressing *E. coli*. In the negative experiment, no LasI-expressing *E. coli* was encapsulated in the trapped droplet. Each data point was averaged by measuring hundreds of single cells.

Having established that the system can be used to deliver signaling molecules to an encapsulated bacterium, the next step was to see a bacterium can sense the presence of another single bacterium placed in a neighboring droplet. This was investigated by encapsulating LasI *E. coli* which can produce OdDHL, in one droplet trapped with the other droplet containing LasR *E. coli*. As shown in Figure 5, after 5 h the fluorescence intensity from 90% of LasR *E. coli* started to increase, indicating LasR *E. coli* sensed the signaling molecules secreted by LasI *E. coli* in the other droplet and produced GFP. By contrast, the negative control experiment showed that in the absence of LasI *E. coli*, no GFP production was observed in the sensor strain. It should be noted that the onset of expressing GFP was 5 h, which was longer than that found in the experiment where droplets containing OdDHL were used (1.5 h). LasI *E. coli* presumably need a period to express OdDHL into the droplet and built up a concentration gradient. Considering the threshold concentration measured before (1 μM), the confinement in a droplet of a single LasI *E. coli* effectively enhanced the process of building up of the concentration of OdDHL in droplets beyond the threshold. In our experiment, the diameter of droplets was 50 μm, which can be regarded as the average communication distance between two bacteria in the trapped double droplets, and the volume of droplet, 65 picoliter, gave rise to an average cell density 1.91 × 10^7^ cells mL^−1^ for single bacterium in one droplet. This result agrees well with the value (46 μm for 10^7^ cells mL^−1^) reported in the literature [21–23]. This one to one communication between two bacteria at single cell level demonstrated the capability of this device as a new technique to study the behavior of single cells in a confined and controlled manner.

## 3. Experimental Section

### 3.1. Materials

*N*-3-(Oxododecanoyl)- l-homoserine lactone (OdDHL) and Fluorinert (FC-40) were obtained from Sigma-Aldrich. EA surfactant (RAS 168-069) was obtained from RainDance Technologies. Poly(dimethylsiloxane) base and curing agent (PDMS, Sylgard 184) was obtained from Dow Corning (Staffordshire, UK).

### 3.2. Device Fabrication

The device was designed using AutoCad (AutoDesk). Photolithographic masks were prepared on transparent plastics (Circuit Graphics, Essex, UK), and standard soft lithographic techniques were used to fabricate devices [16]. Briefly, SU8-2025 photoresist (MicroChem, Warwickshire, UK) was spin-coated to a final film thickness of 50 μm, as measured by profilometry on the finished master (DekTak 150, Coventry, UK). After spinning, the wafer was pre-baked (3 min at 65 °C, then 6 min at 95 °C and finally 3 min at 65 °C), and then exposed to UV light through a dark-field mask on a mask aligner (MJB4, Suss Microtech, Coventry, UK). After post-baking for 1 min at 65 °C and 3 min at 95 °C, the master was developed for 6 min and then hard-baked for 1 min at 170 °C. The master was used to prepare PDMS devices. PDMS base and curing agent (10:1, Sylgard 184) was poured over the master, degassed for 30 min and then baked overnight at 75 °C. The devices were cut and peeled off the master. Access holes for the inlet tubes were punched using a biopsy punch. The PDMS was then exposed to an air plasma for 8 s (Diener Femto plasma asher, Warrington, UK) sealed to a glass microscope slide, and baked overnight at 75 °C. For fluorophilic surfaces, the channels of the device were treated with Aquapel reagent and washed with FC-40. To avoid evaporation of the aqueous phase the PDMS device was sealed in a chamber filled with water for 3 days before experiments.

### 3.3. Droplets Generation

The flow of liquid through the device was driven with Harvard Apparatus 2000 syringe infusion pumps using plastic syringes (B. Braun, Sheffield, UK) connected to polyethylene tubing (Beckman and Dickinson, Oxford, UK). A solution of 1% RainDance EA surfactant (*w*/*w* %) in FC-40 was used as the oil phase in a fluorophilic PDMS device. A flow-focusing configuration was used to generate 50 μm diameter droplets. The size, frequency and speed of the droplets within the device were regulated by controlling the flow rate of the aqueous solutions and oil flow. A typical setup used the combination of 80 μL/h oil phase and 20 μL/h aqueous phase.

### 3.4. Optical Detection

The trapped droplets were observed at 1000 frames per second by movement of the device in a pre-determined pattern using a computer-controlled motorized stage (H117 ProScan II, Prior Scientific, Rockland, MA, USA). The fluorescence images were taken using an EM-CCD camera (Xion+, Andor Technologies, Belfast, UK) connected to an inverted microscope (IX71, Olympus, Southend-on-Sea, UK) operating in epifluorescence mode, with a mercury lamp (U-LH100HG, Olympus) for wide-field illumination and filters (Olympus) to separate the fluorescence excitation and emission light. Photobleaching was minimized by illuminating the droplets only during measurements by using a computer-controlled shutter. The image acquisitions and image analysis was performed using image J and software written in Labview.

### 3.5. Plasmid Construction

Construction of pSB1014 (mCherry expression plasmid): The chloramphenicol resistance cassette from pACYC184 [17] was PCR amplified using primers SP102 (AAAGCCCTTCCGGCTAAATAAATAAATCGGCACGTAAGAGGTTCC) and SP103 (GATGCCGGAAGGGCCGAATTTCTGCCATTCATCC) and cloned into the BglI site within the ApR cassette of pBAD30 [18], thereby disrupting it and generating plasmid pBAD30-cat. The red fluorescent protein, mCherry, was PCR-amplified from plasmid pmCherry-N1 (Clontech) using primers SBP57 (TTTTGGTCTCTTACTTGTACAGCTCGTCCATGC) and SBP61 (TTTTGAATTCTAAATAAAATAAAGGAGGAGTCCCTTATGGTGAGCAAGGGCGAGGAGGA). The PCR product was digested with EcoRI and cloned into the SmaI EcoRI sites of pBAD30-cat to generate plasmid pSB1014 which was able to express mCherry protein in response to l-arabinose.

Construction of pBAD30-lasI: the lasI gene was PCR amplified from *P. aeruginosa* genomic DNA using primers SP22 (GATCCCGGGAGGAGGACAGGGATGATCGTACAAATTGGTCGG) and SP29 (GATAAGCTTTCA TGAAACCGCCAGTCGCT) and cloned into the XmaI HindIII sites of pBAD30-cat. This construct is able to express LasI in response to addition of the inducer, l-arabinose.

### 3.6. Cell Preparation

LasR expressing strain (OdDHL sensing strain): *E. coli* strain DH5α cells harboring pMHLAS and pSB1014 were grown to an A600 nm ~0.85 and diluted to A600 nm ~0.1 with LB-media containing 25 μg mL^−1^ of chloramphenicol, 15 μg mL^−1^ of gentamicin, 0.1 mM IPTG and 0.4 *w*/*v*% l-arabinose and loaded into plastic syringes (B. Braun, Germany, 1 mL) for encapsulation into the device.

The LasI expressing strain (OdDHL producing strain): *E. coli* strain DH5α containing the pBAD30-lasI plasmid was grown to an A600 nm ~0.85 and diluted to A600 nm ~0.5 in LB-media containing 25 μg mL^−1^ of ampicillin and 0.4 *w*/*v*% l-arabinose.

## 4. Conclusions

We have shown, using a double droplet trapping system, that the quorum sensing of single bacterium was observed when it was either exposed to a neighboring pool of signaling molecule, OdDHL, or a droplet containing another single bacterium. Due to the limitation of diffusion, a concentration of 1 μM was found to be the threshold for triggering quorum sensing. The results demonstrated the important role of diffusion, in triggering bacterial quorum sensing and the confinement offered by droplets, in facilitating the build up of concentration beyond the threshold. For droplet-based cell analysis, our approach provides the possibility of studying the communication and interaction between bacteria confined in a small volume at single cell level. By changing contents encapsulated in droplets, this technique might be useful for studying other signaling molecules induced quorum sensing systems or even inter species interaction in the future.
